# Influence of Reaction Parameters on the Catalytic Upgrading of an Acetone, Butanol, and Ethanol (ABE) Mixture: Exploring New Routes for Modern Biorefineries

**DOI:** 10.3389/fchem.2019.00906

**Published:** 2020-01-10

**Authors:** Elham Ketabchi, Laura Pastor-Pérez, Harvey Arellano-García, Tomas Ramirez Reina

**Affiliations:** Department of Chemical Engineering, University of Surrey, Guildford, United Kingdom

**Keywords:** ABE upgrade, reaction optimization, Fe catalyst, long chain hydrocarbons, green chemistry

## Abstract

Here we present a comprehensive study on the effect of reaction parameters on the upgrade of an acetone, butanol and ethanol mixture—key molecules and platform products of great interest within the chemical sector. Using a selected high performing catalyst, Fe/MgO-Al_2_O_3_, the variation of temperature, reaction time, catalytic loading, and reactant molar ratio have been examined in this reaction. This work is aiming to not only optimize the reaction conditions previously used, but to step toward using less energy, time, and material by testing those conditions and analyzing the sufficiency of the results. Herein, we demonstrate that this reaction is favored at higher temperatures and longer reaction time. Also, we observe that increasing the catalyst loading had a positive effect on the product yields, while reactant ratios have shown to produce varied results due to the role of each reactant in the complex reaction network. In line with the aim of reducing energy and costs, this work showcases that the products from the upgrading route have significantly higher market value than the reactants; highlighting that this process represents an appealing route to be implemented in modern biorefineries.

## Introduction

With the increasing need to move toward sustainable energy, many conventional processes have found their usage diminished, especially in the transportation and chemical industries in order to look toward “green” production of fuels and chemicals that are less dependent on fossil fuel. One such “green” production process is bio-refining. Bio-refining incorporates biomass as its feedstock to produce fuels and chemicals of comparable quality to those obtained from conventional petroleum refining (Aresta et al., 2012). One example of this is the production of Acetone, Butanol, and Ethanol (ABE) from sugar fermentation. Furthermore, it has been shown recently that the conversion of ABE to value added products, can be achieved using economically viable catalysts that attain activity and yields similar to those exhibited by noble metal based catalysts (Ketabchi et al., 2019). Using the standard ratio of ABE (3:6:1) as reactants, under the conditions of high temperatures and pressures, they undergo three main steps of a complex reaction mechanism consisting of the initial dehydrogenation of the primary alcohol, a catalyzed aldol coupling reaction, which then continues with the hydrogenation of an α, β-unsaturated aldehyde (Chakraborty et al., 2015). This reaction requires catalysts that can fulfill the requirements of each step in the mechanism, therefore, a multifunctional catalyst containing both acidic and basic sites have proven to ensure the chain elongation of the starting reactions, producing added value products (Di Cosimo et al., 1998; Kikhtyanin et al., 2017, 2018). Using transition metals for the production of value added chemicals and products has received an increased attention due to its economic favorability and success (Qiu et al., 2019).

For our seminal work, we examined 3 different transition metal catalysts, finding not only promising results in terms of the production of valuable long chain hydrocarbons, but also offering a suitable alternative for cost reduction when compared to noble metals which is commonly used for this process (Anbarasan et al., 2012; Marcu et al., 2013; Morvan et al., 2014; Sreekumar et al., 2014; Onyestyák et al., 2015a; Goulas et al., 2017). Among the different tested systems the multicomponent Fe/MgO-Al_2_O_3_ catalyst displays the best activity/selectivity performance (Ketabchi et al., 2019). This catalyst presents ideal acid-base properties which are essential for this process. In particular, Fe promotes condensation reactions, having stronger Lewis basic character compared to the other two transition metals tested in our previous work, Ni/MgO-Al_2_O_3_ and Cu/MgO-Al_2_O_3_, respectively (Ketabchi et al., 2019). It also demonstrates a favored surface interaction with the reactions through alkoxy structures leading to higher conversions (Unnikrishnan and Narayanan, 1999).

After choosing the best performing catalyst, to enhance this further, it is important to optimize the reaction conditions to achieve the best possible results. Hence, in this study, a number of reaction parameters such as temperature, catalytic loading, reaction time, and reactant molar ratio have been varied, to present a systematic study. The effects that each permutation has on the desired product yield and reactant conversion, will be considered. Simultaneously, a reusability study of the Fe-based catalyst has also been carried out to prove the practicality, recyclability, and success of the selected catalyst followed by a preliminary economic study to evaluate the market value of the products.

Under these premises, the main focus of this work, therefore, is the optimisation of ABE upgrading process using our engineered multicomponent catalyst. At the same time we aim to inspire the catalysis and bio-refining community to explore this route as a potential economically viable pathway to be implemented in modern bio and hybrid refineries.

## Experimental

All materials were purchased from Sigma-Aldrich, unless stated otherwise, and were used as received.

### Catalyst Preparation

The support and final catalyst were prepared by wet impregnation method reported in our previous work (Ketabchi et al., 2019). A typical synthesis involved the impregnation of mesoporous γ-Al_2_O_3_ (Sasol) with magnesium nitrate hexahydrate, Mg(NO_3_)_2_·6H_2_O, in a rotary evaporator for 1 h before the removal of the solvent. This suspension was then dried overnight in an oven and then calcined in a furnace for 12 h at 700°C, 10°C/min ramp, resulting in the MgO–Al_2_O_3_ support. This support was then impregnated once more with a solution of iron (III) nitrate nonahydrate, Fe(NO_3_)_3_·9H_2_O, followed by drying and calcination using the previous conditions. The resulting catalyst was named Fe/MgO-Al_2_O_3_.

### Catalyst Characterization

X-ray diffraction (XRD) was carried out on the catalysts using a PANalytical X'Pert3 Powder diffractometer with Cu-Kα radiation at room temperature and 2θ angle between 10 and 90° at 40 kV and 30 mA. The pattern obtained from each sample was further processed using X'PertHighscore Plus software and plotted in Origin 2018b.

Thermogravimetric analysis (TGA) was carried out on the spent catalysts in a TGA/SDTA851e/LF/1600 instrument (Mettler Toledo) connected to a mass spectrometer (TGA-MS). Samples were exposed to air from room temperature to 900°C at 5°C min^−1^.

Characterization and further analyses on the calcined and reduced sample of the catalyst can be found in our previous patented work (Ketabchi et al., 2019).

### Catalytic Activity

All experiments of ABE upgrading were performed in a pressure vessel/Parr reactor (Parr Series 5500 HPCL Reactor and a 4848 Reactor Controller). The reactor was purged with N_2_ to ensure an oxygen-free atmosphere. The batch reactor was filled with various amounts of acetone, butanol and ethanol (Sigma-Aldrich) and the reduced catalyst (loading dependent on permutation). A range of temperatures (200–300°C), reaction times (3–18 h), catalyst loading (0.2–0.5 g) and a variety of reactant ratios were tested to identify their respective effect and to tune the reaction toward the desired outcome. Changes to the conditions were tested individually, to be certain of the effect each change had on the overall system.

The molar ratio of the reactants was varied over four experiments, including the typical molar ratio of produced ABE from sugar fermentation (Acetone = 22.5 ml, Butanol = 55 ml, and Ethanol = 22.5 ml) (Cabezas et al., 2019), while keeping temperature, catalyst amount and reaction time constant.

For the recyclability study, two sets of reactions were carried out. The first set involved a reaction using the typical molar ratio of ABE (Cabezas et al., 2019), 0.35 g of catalyst at 300°C. The spent catalyst from this reaction was dried and then used in the same reaction conditions without reduction. The second set involved the same conditions, but with the addition of a pre-reduction step before the spent catalyst was reused.

The gas chromatograph-mass spectrometer (GC-MS) used to quantify and identify the reaction products, was fitted with the same column as the GC-FID and operated using the same temperature program.

After all experiments, the catalysts were recovered through filtration and the liquid products were analyzed in an Agilent HP6890 gas chromatograph (GC), equipped with a DB-5 Capillary Analytical column and fitted with a flame ionization detector (FID).

Conversion was calculated using Equation 1. Yield to liquid products is defined as the percentage of carbon moles transferred from the initial ABE mixture to the liquid products obtained. This was calculated by dividing the amount of carbon moles in each fraction by the total carbon moles of the ABE loaded into the reactor (Equation 2).

(1) $$\begin{array}{r} ABE\;Conversion\;\left(\%\right)=\frac{\;C_{Initial\;ABE}-C_{Unreacted\;ABE}}{\;C_{Initial\;ABE}}\times 100 \end{array}$$

(2) $$\begin{array}{r} Yield\;\left(\%\right)=\frac{C_{Product}}{C_{Initial\;ABE}}\;\times 100 \end{array}$$

Where C is the number of carbon moles of reactants or product.

## Results and Discussion

In this section, the catalytic activity is investigated individually for each reaction so that the effect of each parameter can be determined so to ultimately identify the most suitable reaction conditions to produce the desired products. Therefore, each section hereafter will only discuss the variations found in the spent sample as well as its comparison with the reduced sample. Information regarding pre-activity of the catalyst can be found in our previous work (Ketabchi et al., 2019).

It should be noted that only the liquid products are considered in our study since they are the most important product in terms of chemical market opportunities.

### Effect of Temperature

To investigate how the process is affected by operational temperature, a series of experiments conducted at different temperatures between 200 and 300°C (300°C is chosen as the reference temperature as this value is used in our patent) were undertaken. These experiments maintained catalyst loading (0.5 g), reactant ratio (A:B:E 3:6:1) and the reaction time (18 h) adapted from literature (Di Cosimo et al., 2000; Alipour et al., 2014). The constant conditions of the reaction were chosen according to our previous work and to allow for comparison. Lower temperatures from the reference temperature are chosen, not only to explore what effect different temperatures would have on this reaction, but to do so in a cost-effective way. Making this process economically favorable in any aspect would be extremely beneficial and attractive for both the industry and research as the conventional route hinders commercialization due to large expense burdens. The catalytically related expenses were heavily reduced with our previous work and now with the investigation of the effect of lower temperatures, the same aim is pursued.

**Figure 1:**
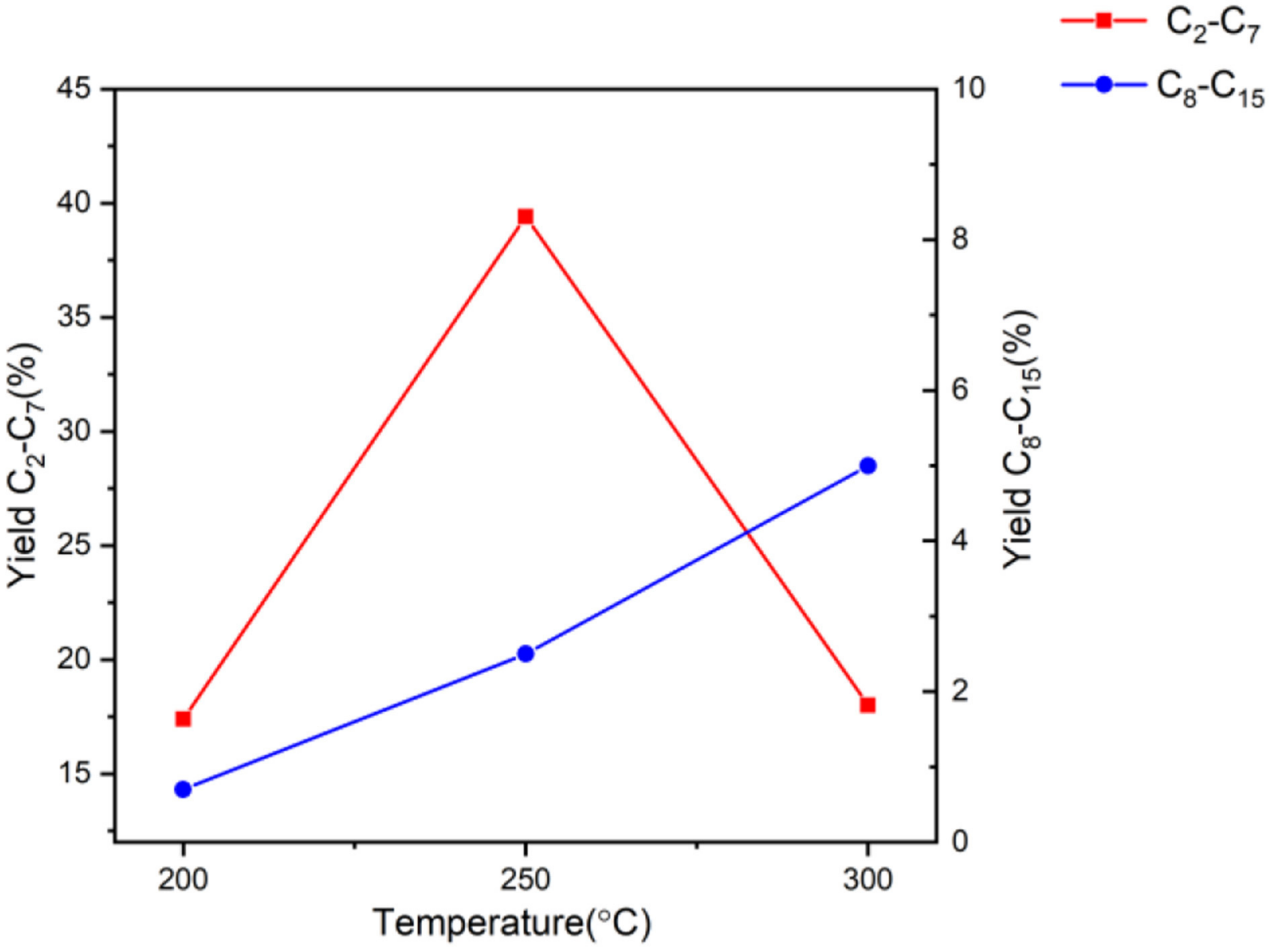
Yield of products in the range of C_2_-C_15_ using the Fe catalyst at various temperatures keeping the amount of catalyst, reaction time, and reactant ratio constant.

Presented below are the catalytic activity results, displaying conversion and yield calculated via Equations 1 and 2. The performance of the catalyst in this reaction is shown in Tables 1, 2 as well as Figure 1.

**Table 1:** Conversion of the reactants to products at various temperatures using the Fe catalyst with constant catalyst amount, reaction time, and reactant ratio.

**Temperature (^**°**^C)**	**Conversion (%)**		
	**Acetone**	**Butanol**	**Ethanol**
200	99	–	95.5
250	99.6	–	95.9
300	99.6	95.9	95.2

**Table 2:** Yield of products having highest concentration at 200, 250, and 300°C using the Fe catalyst with constant amount, reaction time and reactant ratio.

**Temperature (**^****°****^**C)**		**200**	**250**	**300**
Yield (%)	2-Propanol (C_3_)	6.45	10	6
	Butanal (C_4_)	0.7	1	2
	n-Butylacetate (C_6_)	–	0.2	–
	2-Heptanone (C_7_)	3.4	3.4	3.8
	2-Heptanol (C_7_)	–	0.6	0.64
	3-Hepten-2-one (C_7_)	–	0.53	0.5
	Butylbutyrate (C_8_)	–	0.33	0.4
	Isophorone (C_9_)	–	–	0.34
	6-Undecanone (C_11_)	–	–	0.4

Table 1 shows the conversion of each reactant in each experiment. High conversions of acetone and ethanol are achieved for all temperatures in this range. This shows the suitability and success of the multicomponent Fe catalyst for this specific reaction. However, butanol conversion could not be calculated using Equation 1 in the experiments of 200 and 250°C; at lower temperatures, shorter reaction times and lower catalyst loading, there is more butanol produced than the initial amount used [due to the nature of the reaction as butanol is both a reactant and a product, as explained in our previous work (Ketabchi et al., 2019), in the acetone and ethanol reaction, also demonstrated further on in Figure 11 (Onyestyák et al., 2015a)]. This means butanol has a required temperature, time and catalyst/reactant ratio to act as an intermediate to further convert to longer chain hydrocarbons. However, the absence of butanol conversion in Table 1 for lower temperatures does not mean that they have not converted. It simply determines that the amount of butanol intermediates produced from acetone and ethanol are more than the amount of reactant butanol.

Beyond the overall conversion levels the yield to added value products is more relevant in this case. Figure 1 shows the effect of temperature on total products obtained with respect to carbon number. It can be seen that with the increase of temperature, the yield toward longer chain hydrocarbons increases, including alkene compounds (Nahreen and Gupta, 2013), which is justified by the nature of the reaction network. Thus, at lower temperatures, the production of shorter chained hydrocarbons is favored and was noted to include such as C_5_ or C_6_ and ether compounds. Furthermore, subsequent dehydration of the intermediates occur at higher temperatures using the acidic sites of the catalyst, leading to aldol condensation reaction producing the alkene materials found (Olcese and Bettahar, 2013). An increase in the lower carbon number range (C_2_-C_7_) can be seen when increasing the temperature from 200 to 250°C, which is then followed by a decrease when testing the reaction at 300°C. This is again due to the nature of the reaction network that starts with the production of shorter chain hydrocarbons, followed by high temperature aldol condensation, shifting the yield toward C_8_-C_15_. This series of condensations consumes the shorter chain hydrocarbons and explains the data trend seen in Figure 1 (Di Cosimo et al., 2000; Cabezas et al., 2019).

Table 2 presents the yields of the significant products identified. There is a plethora of organic compounds present in the liquid phase of these samples ranging from C_2_ to C_15_ with the significant products, namely: 2-propanol, butanal, n-butylacetate, 2-heptanone, 2-heptanol, 3-hepten-2-one, butyl-butyrate, isophorone, and 6-undecanone being the biggest portion of the liquid product obtained in the reaction cycle.

As established in the results of the test at 200°C in Table 2, the most yielded product is 2-propanol, though limited quantities of 2-heptanpone are also present. At 250°C, the reaction produces higher yields of 2-heptanone while also yielding a range of products that are not present at 200°C, such as butylbutyrate. Similarly, 6-undecanone can be seen when conducting the experiment at 300°C but is not present at lower temperatures. Comparing the amount of significant product yield, as expected and explained previously, the increase in longer chain hydrocarbons can be seen with temperature, finding also a number of more complex organic compounds only produced as temperature increases. For 2-propanol, produced by the reaction of ethanol and acetone (Onyestyák et al., 2015a), an increase in yield has occurred between 200 and 250°C that details the progression of the reaction network explained in more detail in our patent (Ketabchi et al., 2019). However, this decreases at 300°C, demonstrating that the shorter chain hydrocarbons are condensing.

**Figure 2:**
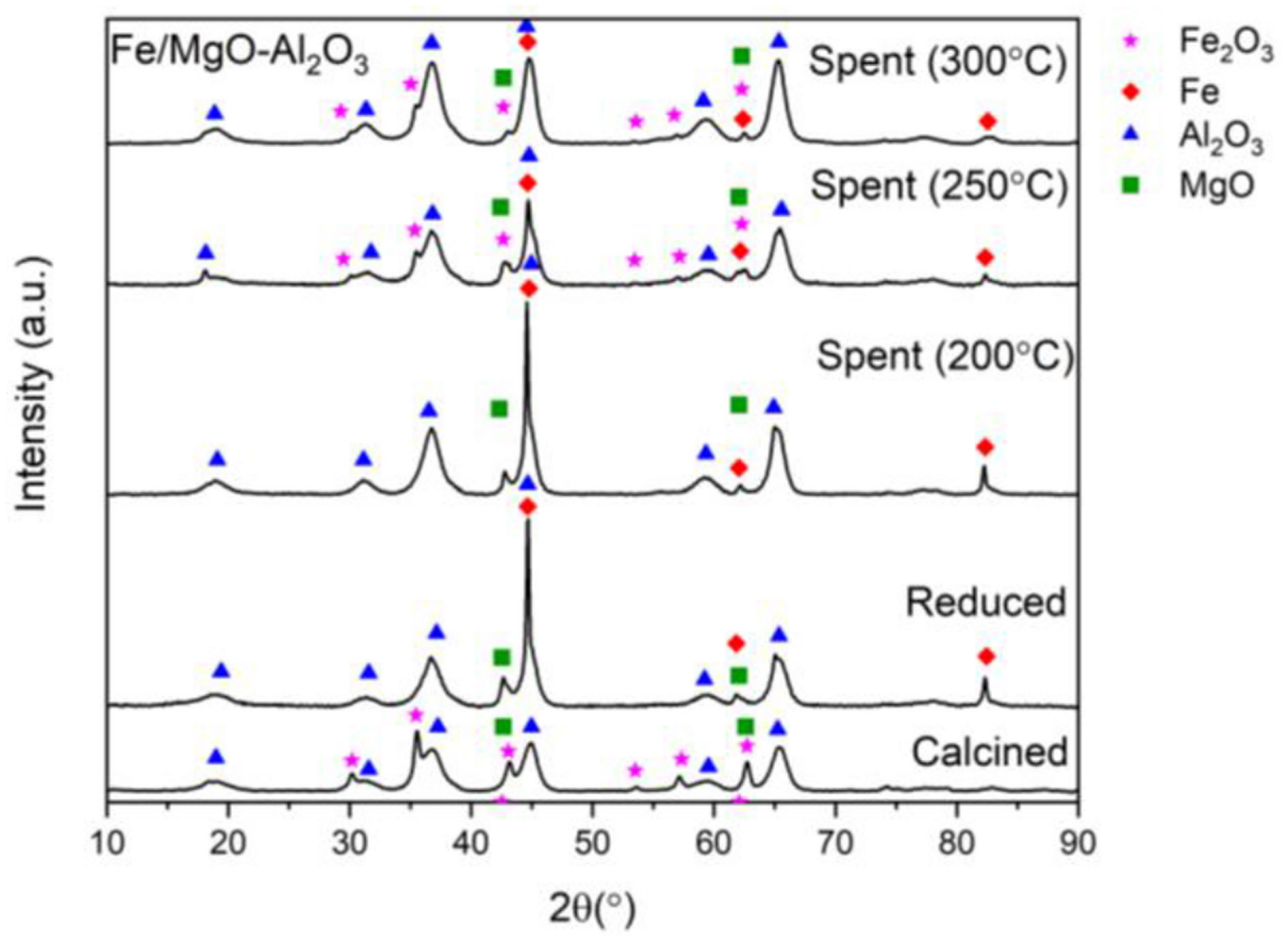
XRD of fresh, active, and spent Fe catalyst at different reaction temperatures.

Presented are the XRD characterization results in Figure 2 for the catalyst at all stages of experimentation (calcined, reduced, and spent) with emphasized focus on the spent sample, alongside Thermogravimetric analysis (TGA) for each spent sample (Figure 4).

Figure 2 contains the XRD patterns of the calcined, reduced and spent Fe-based catalyst after reaction at 200, 250, and 300°C. Starting with the 200°C experiment, the spent sample diffractogram in Figure 2 shows that the significant Fe peak at 44.7°, remains consistent compared to the reduced catalyst showing that no sintering has occurred (Mendez-Garza et al., 2013).

Moving on to the 250°C experiment, the corresponding metallic Fe peaks are smaller compared to peaks from the reduced sample, that could be due to loss of active phase through oxide formation at 2θ = 35.4, 43.1, and 62.6°. These minor peaks are identified as Fe_2_O_3_ (Wang et al., 2014; Dasireddy et al., 2018).

**Figure 3:**
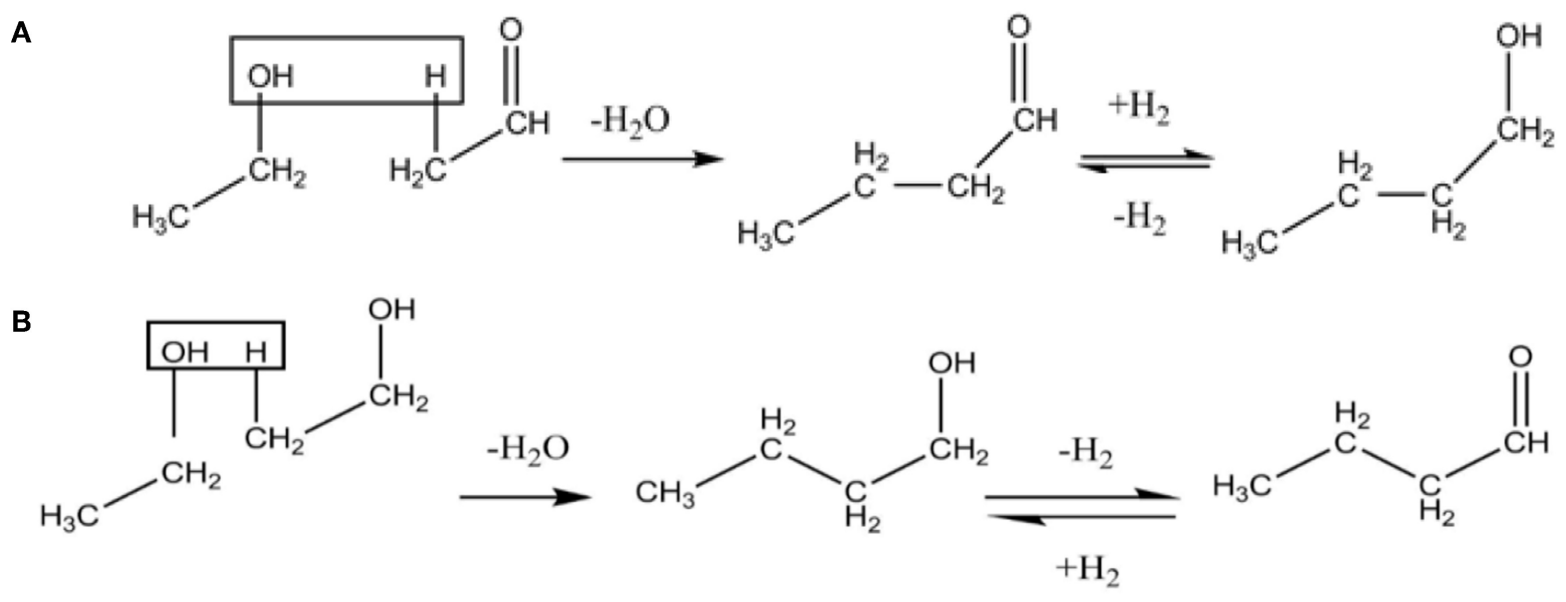
Condensation of Ethanol with acetaldehyde **(A)** followed by dimerization of Ethanol **(B)** adapted from Ndou et al. (2003).

In order to explain the emergence of these iron oxide peaks, the reaction conditions and compounds present as a result of many reactions occurring should be considered. The reaction network involves ethanol dehydrogenation at the basic site of the catalyst producing acetaldehyde, which is then followed by the dimerization of ethanol or condensation of ethanol with acetaldehyde, is carried out through hydrogen abstraction from a β-C atom to produce water (Figure 3).

Due to the presence of water (Figure 3) and high temperatures, partial oxidation of metallic iron to produce the Fe_2_O_3_ can possibly occur which also shows its presence in the post reaction XRD patterns. However, the emerged peaks are very small concluding that there is only a limited degree of oxidation. The speculated conversion of Fe to Fe_2_O_3_, is further supported by both the increase in Fe_2_O_3_ and decrease in Fe (44.7°) peak intensity, following the 250°C experiment. This has also been checked by EDX that the Fe peak reduction is not due to leaching but due to Fe_2_O_3_ formation as the amount of Fe has been consistent in both reduced and spent samples. This occurrence is also seen in the 300°C experiment, with the Fe peak at 44.7° also decreasing in intensity, though to a lesser degree than seen at 250°C.

**Figure 4:**
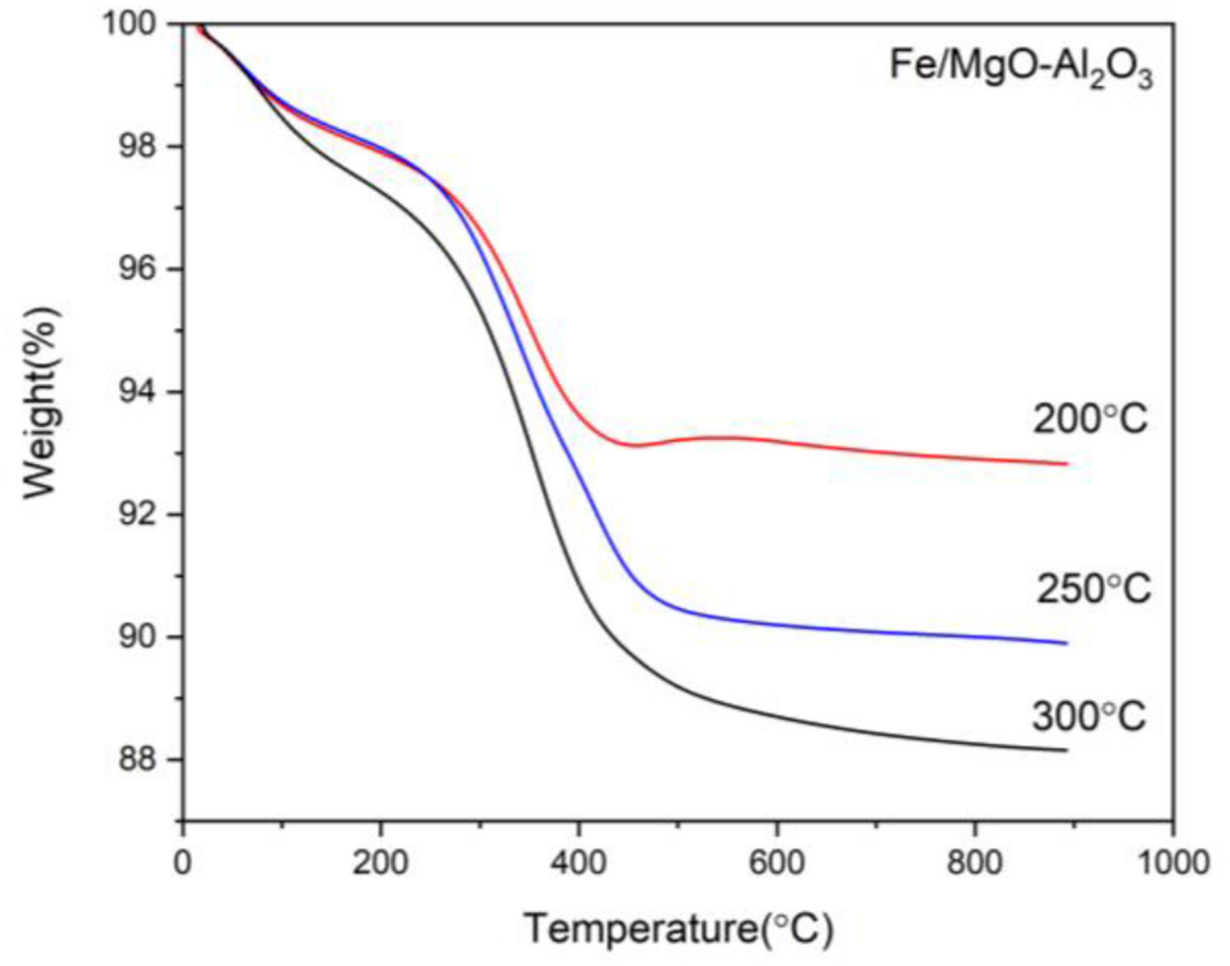
TGA curves for the spent Fe catalyst at various reaction temperatures.

TGA of the spent catalysts are displayed in Figure 4. Herein, there is an immediate weight loss due to the presence of water in the samples, between room temperature (RT) and 150°C, followed by carbon loss between 150 and 350°C as well as 350 and 500°C that is attributed to organic compounds attached to the sample after reaction indicating reaction progress and success. The second zone is speculated to be regarding lighter organic compounds attached while the last zone is for the heavier organic compounds. It can be seen that the highest loss in the 150–350°C zone is attributed to the 250°C experiment which is justified by the yield results as well. Moving to the next zone, as expected, the higher the temperature of the reaction, the more heavier organic compound is deposited on the catalyst displaying higher activity of the sample: 5.3, 8.3, and 9.4 wt% for the 200, 250, and 300°C experiments, respectively.

Ultimately, identifying the optimal temperature for this reaction purely depends on the desired products. It is clear that this reaction is favored at higher temperatures (300°C in this case) with the added incentive of fully converting butanol to the long chain hydrocarbons. However, if the shorter chain products are desired, operating the process at 250°C produces more desirable products than at 200°C. Even though that there is the possibility of being able to produce longer chain hydrocarbons with 250°C using longer reaction times, this would defy the purpose of this study which is looking into producing the favorable results comparable to our previous work while reducing the time, energy and material required.

### Effect of Reaction Time

This series of experiments were undertaken varying the reaction times from 3–18 h, while maintaining the catalyst loading (0.5 g), reactant ratio (A:B:E 3:6:1) and the temperature (300°C). These reaction times are used to pursue one of the aims of this study, being the investigation of the ability to achieve comparable results with less time, energy and material and to optimize the reaction according to our previous study.

As shown in Table 3, all reactions detailed high conversion of acetone and ethanol. However, as noted previously, due to butanol being both reactant and product in these reactions, it was not possible to calculate conversion. It was noted that at lower reaction times, the network (detailed in our patent, Ketabchi et al., 2019) does not fully proceed; only fulfilling the initial steps of the process that includes the production of butanol as an intermediate that leads to the surplus of butanol in the liquid products. Since distinguishing between the amounts of intermediates produced and the unreacted reagent is not possible, this produces negative conversions; data regarding conversion in this case is omitted.

**Table 3:** Conversion of the reactants to products at various reaction times using the Fe catalyst with constant catalyst amount, temperature, and reactant ratio.

**Reaction time (h)**	**Conversion (%)**		
	**Acetone**	**Butanol**	**Ethanol**
3	98.2	–	94
6	99.6	–	94
9	99.3	–	94
18	99.6	95.9	95.2

**Figure 5:**
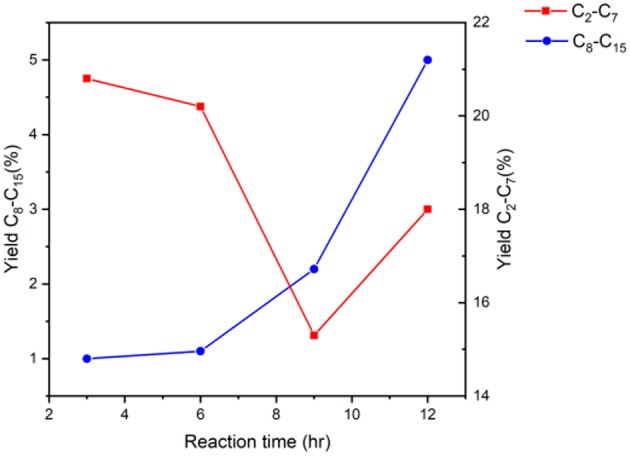
Yield of products in the range of C_2_-C_15_ using the Fe catalyst at various reaction times keeping the amount of catalyst, temperature, and reactant ratio constant.

Figure 5 displays the effect of reaction time on the yield of the targeted group of products. It is clear that with the increase of reaction time, the reaction moves toward producing longer chained hydrocarbons as it has more time to proceed. Starting with mono alkylation, with longer reaction time, mono alkylation transitions to double alkylation resulting in longer carbon chain products. Moving from 3 to 6 h, there is no significant effect to either hydrocarbon range. However, when comparing 6 to 9 h, there is a significant drop in shorter chain hydrocarbon yield that coincides with double the yield of long chain hydrocarbon, clearly suggesting the link between chain growth from condensation reactions and reaction time. On the other hand, there is a slight increase in short chain hydrocarbon yield when we reach 18 h of reaction, comparted to 9 h. This suggests that the reaction has had sufficient time to improve the yield of short chain hydrocarbons, while further alkylating the intermediates to longer chain hydrocarbons.

Table 4 shows the most prominent products obtained over the course of these reactions. Similar to the temperature study, 2-propanol decreases with time suggesting that this is a crucial intermediate alcohol in the subsequent condensation reactions toward longer chain hydrocarbons. However, we see an increase in the production of Butanal after 18 h, which shows that the reaction was not able to produce as much at shorter reaction times. Butanal is obtained via ethanol dimerization via a three-step reaction, shown in Figure 3. This result confirms that this reaction requires time to produce butanal.

**Table 4:** Yield of products having highest concentration with various reaction times using the Fe catalyst with constant amount, temperature, and reactant ratio.

**Reaction time (h)**		**3**	**6**	**9**	**18**
Yield (%)	2-Propanol (C_3_)	7.3	8	8	6
	Butanal (C_4_)	0.9	0.9	0.7	2
	2-Heptanone (C_7_)	4.3	5	4.4	3.8
	2-Heptanol (C_7_)	0.6	0.6	0.6	0.64
	3-Hepten-2-one (C_7_)	0.5	0.5	0.5	0.5
	Butylbutyrate (C_8_)	0.2	0.32	0.4	0.4
	Isophorone (C_9_)	0.06	0.21	0.3	0.34
	6-Undecanone (C_11_)	–	–	0.3	0.4

Three compounds, 2-heptanone, 2-heptanol, and 3-hepten-2-one, are present in the products throughout the duration range, demonstrating the possibility of these products being obtained within the first 3 h of the reaction. This could be due to the temperature chosen for these reactions (300°C), as the previous study found none of these organic compounds at lower temperatures, even after 18 h of reaction. The remaining products presented in the table show an increase or even appearance with longer reaction time since the reaction has more time to progress to longer chain hydrocarbons in this case.

When comparing the results of the 9 h experiment to the 18 h experiment, the long chain hydrocarbon yield improved only marginally. Therefore, it can be inferred that a 12 h study would not have revealed any noteworthy difference in this trend. Although the fluctuations of product yields are minor the data serves to indicate a trend which we have analyzed. This nevertheless emphasizes the impact of temperature on these reactions, demonstrating that high temperatures are favored.

**Figure 6:**
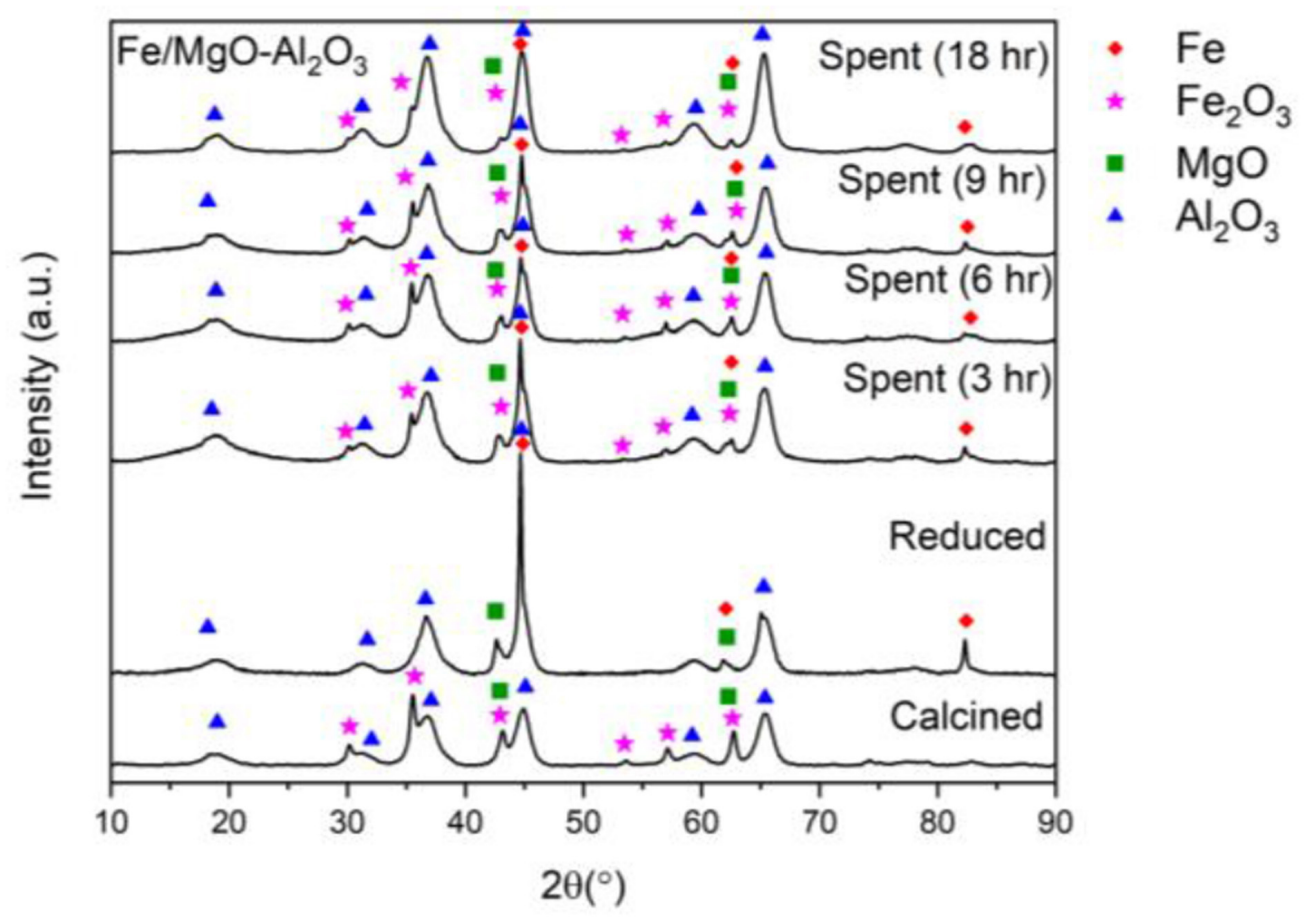
XRD of fresh, active, and spent Fe catalyst at different reaction times.

Figure 6 depicts the XRD patterns of the calcined, reduced and spent after 3, 6, 9, and 18 h. As previously mentioned in the temperature study, the calcined and reduced sample of the catalyst show the expected peaks of Fe_2_O_3_, MgO and Al_2_O_3_ in the calcined sample as well as metallic Fe peaks emerging in the reduced sample (Malinowski et al., 2009; Strassberger et al., 2013; Wang et al., 2014; Dasireddy et al., 2018). However, when observing the spent catalysts, there is a decrease in the intensity of the Fe peak at 44.7° that can be seen in all spent samples, compared to the reduced sample. This decrease could be due to catalyst oxidation (Fe to Fe_2_O_3_), as can be seen in the diffractogram.

The same trend can be seen with the increase of reaction time having slight peak intensity reductions for Fe at 44.7°, with the least intense peak corresponding to the maximum reaction time of this study (18 h). Similar to the temperature study, the appearance of Fe_2_O_3_ peaks is thought to be due to partial oxidation under hydrothermal conditions. The Fe peak is still consistently present in all spent samples, demonstrating that the formation of Fe_2_O_3_ and the presence of water does not affect the yield and conversion, which is also supported by literature (Alsawalha, 2019).

**Figure 7:**
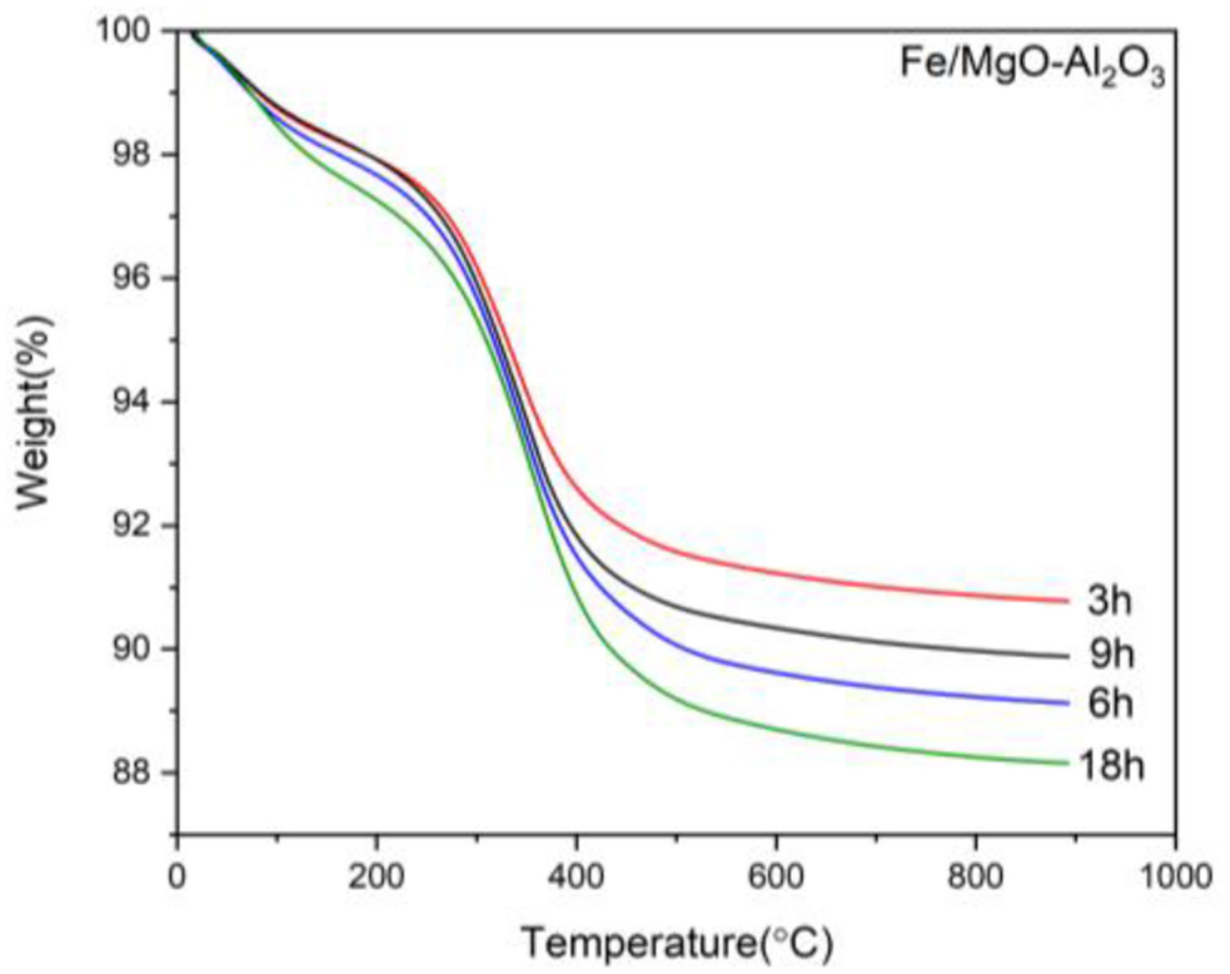
TGA curves for the spent Fe catalyst at various reaction times.

The TGA results in Figure 7 displays the same initial water weight losses as before (RT-150°C). After 150°C there is also a similar weight loss attributed to the carbon loss associated with organic compounds attached to the spent samples, the weight loss increasing with reaction time.

As previously noted, there are two clear zones after the water loss, attributed to light and heavy organic compounds attached, 150–350°C and 350–500°C, respectively. It can be seen that at lower reaction times, a higher amount of light organic compounds is attached while with higher reaction times, higher heavy organic compounds are attached. It must be noted that in the case of 6 to 9 h, there is a minor decrease that is not following the expected trend. Nevertheless, the carbon difference between the two samples is negligible (0.4% increase).

Overall, it is quite clear that longer reaction time favors long chain hydrocarbons, which also ensures that the reaction is almost entirely complete, judging from the conversion and yield data.

### Effect of Catalyst Loading

The third series of experiments was conducted with a varied catalyst loading, from 0.2 to 0.5 g, maintaining the reaction time (18 h), reactant ratio (A:B:E 3:6:1) and the temperature (300°C).

After identifying in our previous paper that this catalyst is cost-effective, it has proven to produce added value products at a comparable or even better level than noble metal catalysts (Marcu et al., 2013). However, if we are still able to achieve remarkable results with less catalyst, this would be tremendously advantageous as our aim is to optimize the reaction while also finding pathways to reduce costs simultaneously.

Observing the conversion values for the reactants found in Table 5, again, the butanol conversion of this set of reactions was impossible to calculate, owing to reasons discussed earlier. However, it is evident that with the increase of catalyst, the reaction has progressed in order to both consume butanol as a reactant while also producing it as an intermediate, then using this intermediate to yield the intended products. This can be seen by the successful calculation of butanol conversion demonstrating there is less unreacted butanol than the initial amount used. Although butanol conversion values for 0.2 and 0.35 g of catalyst were inconclusive, the data suggested an improvement when more catalyst was used which could be explained by the increased availability and concentration of active sites promoting the further conversion of intermediate butanol to final products. This progression will also be presented in the yield results.

**Table 5:** Conversion of the reactants to products using the Fe catalyst at varied amounts with constant reaction time, temperature, and reactant ratio.

**Catalyst loading (gr)**	**Conversion (%)**		
	**Acetone**	**Butanol**	**Ethanol**
0.2	99.5	–	94
0.35	99.6	–	95.1
0.5	99.6	95.9	95.2

**Figure 8:**
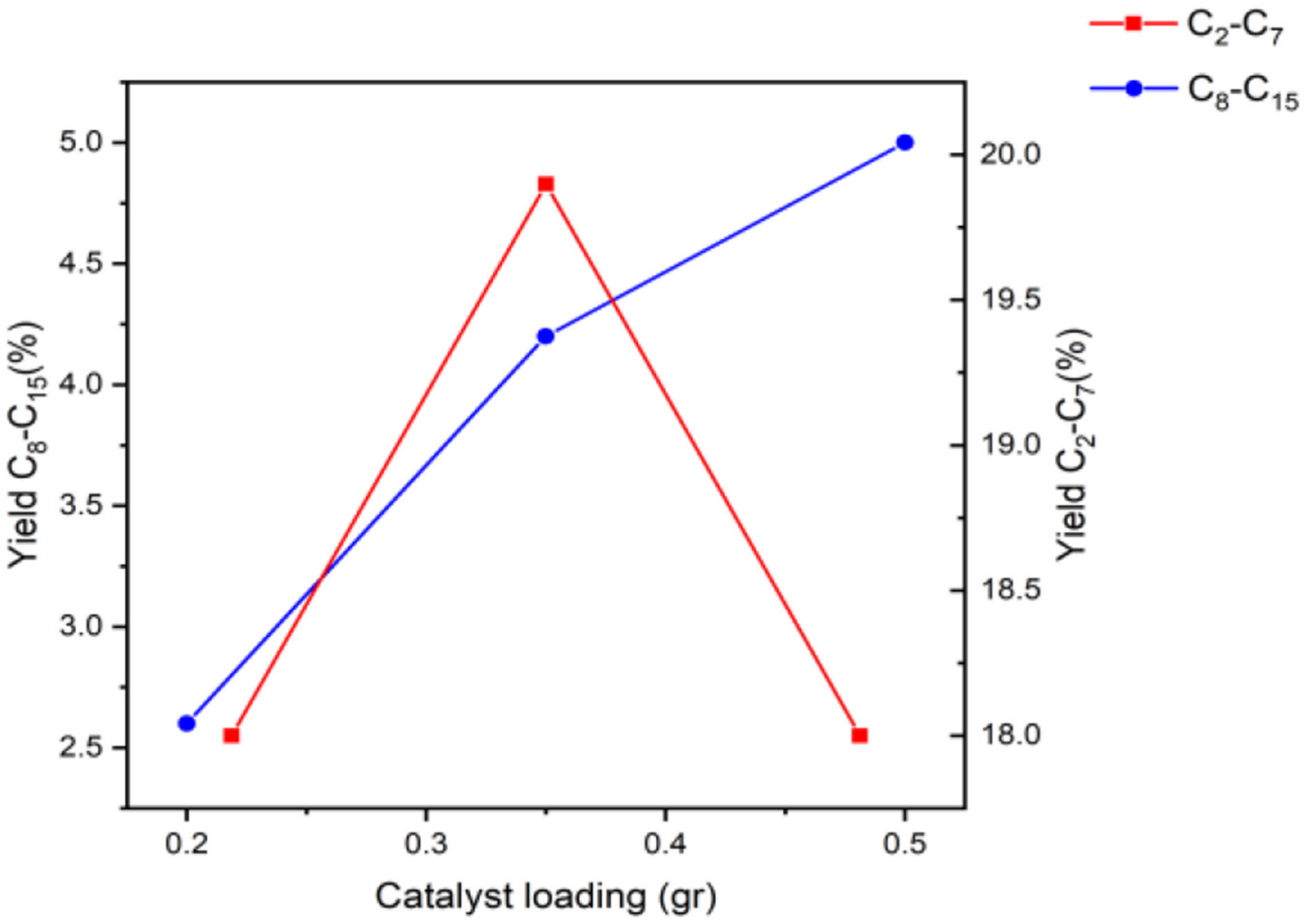
Yield of products in the range of C_2_-C_15_ using the Fe catalyst at various amounts keeping the reaction time, temperature, and reactant ratio constant.

The yield data presented in Figure 8 exhibits a clear increasing trend in long chain hydrocarbon yield with the increase of catalyst loading. Furthermore, a minor increase in the yield of shorter chain hydrocarbons is found when transitioning from 0.2 to 0.35 g, due to the progression of the reaction network. Interestingly, this increase is in the top end of the short hydrocarbon range (C_7_), suggesting that a 0.15 g increase in catalyst loading substantially accelerates the rate of production of longer hydrocarbon product. Concerning the short chain hydrocarbons, this increase specifically effected 2-heptanone and 3-hepten-2-one both having 25% increase. Table 6 also details a number of the long chain hydrocarbons that benefitted from this increased catalytic loading: butylbutyrate, isophorone, and 6-undecanone.

**Table 6:** Yield of products having highest concentration with various catalyst amounts using the Fe catalyst with constant reaction time, temperature and reactant ratio.

**Catalyst amount (gr)**		**0.2**	**0.35**	**0.5**
Yield (%)	2-Propanol (C_3_)	7	7	6
	Butanal (C_4_)	0.9	0.9	2
	2-Heptanone (C_7_)	4	5	3.8
	2-Heptanol (C_7_)	0.6	0.6	0.64
	3-Hepten-2-one (C_7_)	0.4	0.5	0.5
	Butylbutyrate (C_8_)	0.2	0.4	0.4
	Isophorone (C_9_)	0.1	0.33	0.34
	6-undecanone (C_11_)	0.1	0.4	0.4

With this addition, the yielding of butylbutyrate has doubled, isophorone has tripled and 6-undecanone has quadrupled. This shows that the increase of active sites in this transition has specifically pushed the reaction toward a significant increase in the long chain hydrocarbon range since the biggest change is concerning C_8_, C_9_, and C_11_. However, when considering the increase from 0.35 to 0.5 g, an overall increase in the two groupings of hydrocarbons was found, but no specific product benefitted as drastically as the previous transition.

**Figure 9:**
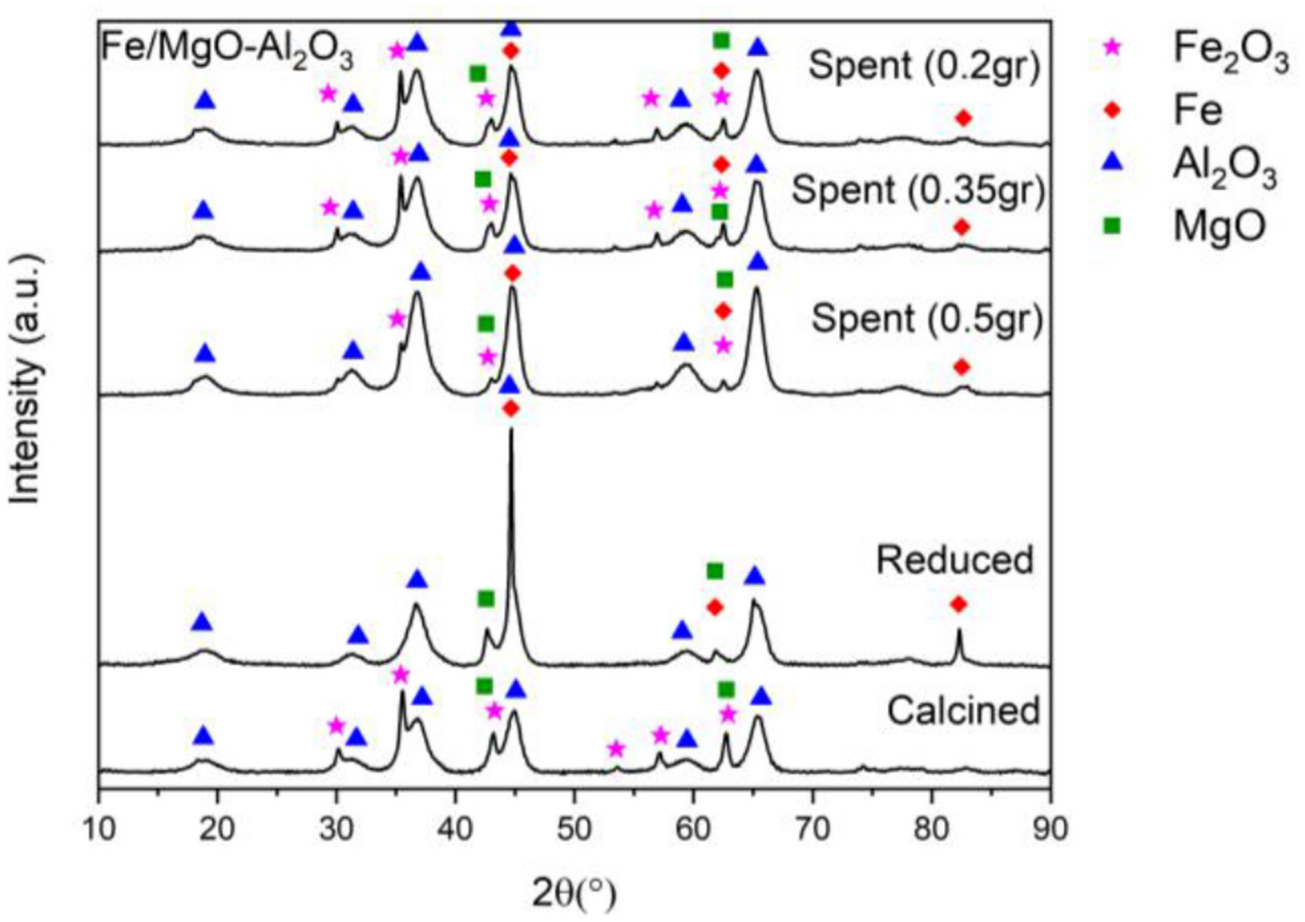
XRD of fresh, active, and spent Fe catalyst at different amounts.

Figure 9 presents the XRD patterns for the spent samples of the reaction when using 0.2, 0.35, and 0.5 g of catalyst alongside the calcined and reduced sample XRD patterns for comparison. Similar to the results discussed above, there is a reduction in the peak intensity of Fe at 44.7° in the spent samples compared to the reduced sample that could be attributed to the formation of Fe_2_O_3_. Between the three spent samples, a decrease in the intensity of the lowest catalyst loading (0.2 g) can be seen. Consequently, at 35.4°, the Fe_2_O_3_ peaks that are present are more intense at lower catalyst loadings, which could be due to the fact there is more oxide formed due to the limited amount of active site when using 0.2 and 0.35 g catalyst, compared to the situation where 0.5 g of catalyst is used. This could in turn be affected by increased competition for the active sites when using less catalyst, causing more rapid oxide formation.

**Figure 10:**
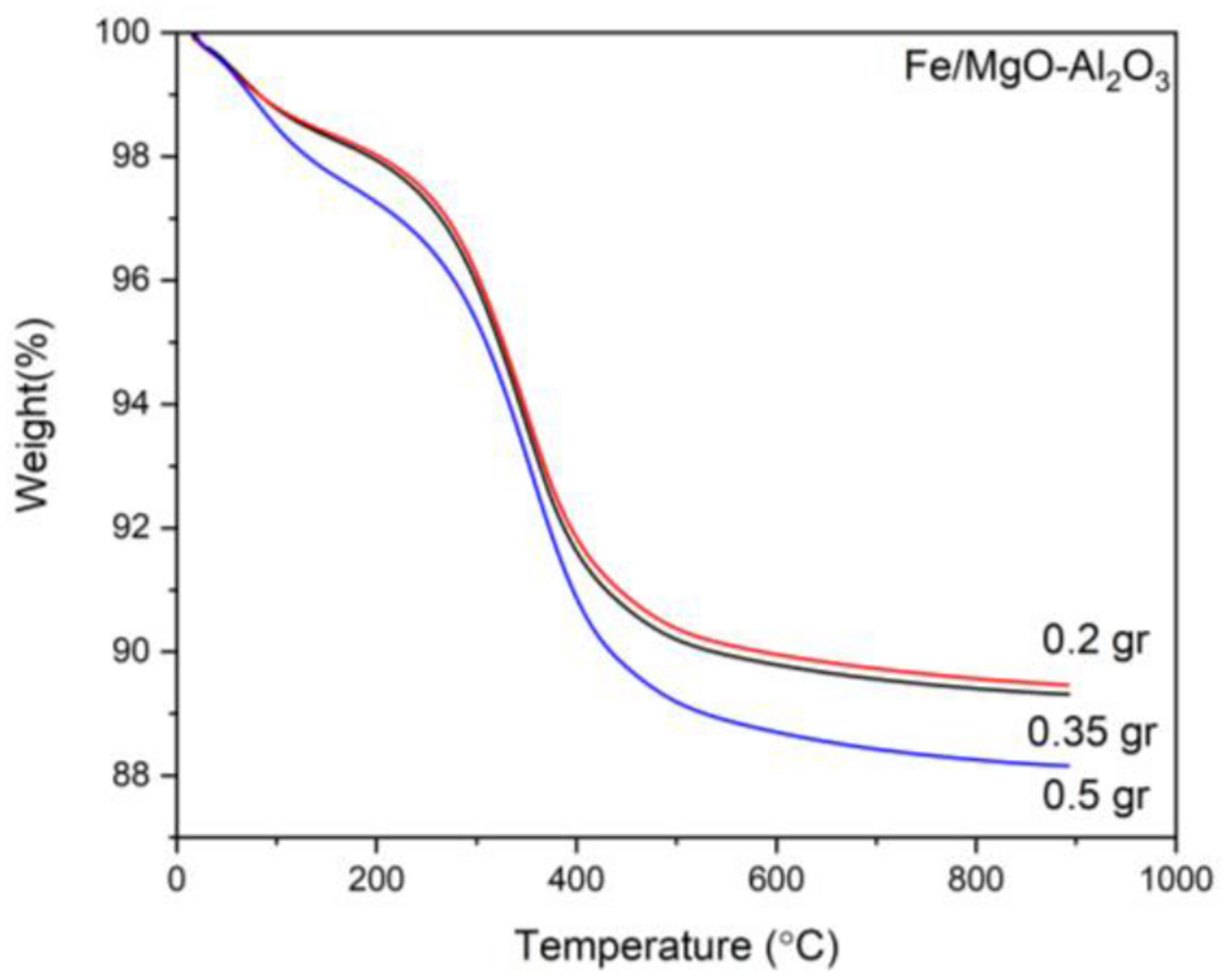
TGA curves for the spent Fe catalyst at various amounts.

The TGA (Figure 10), depicts loss of water from RT to 150°C followed by a weight loss corresponding to carbon deposits from organic compounds in two zones. It is clear that the first zone ascribed to lighter organic compounds has the highest loss when using the least amount of catalyst while the second zone regarding heavier organic compounds has the highest loss when using the most amount of catalyst. It can be seen that there is an increase in the amount of carbonaceous loss with the increase of catalyst loading, most likely due to increased conversion at higher loadings leading to more attached organic compounds, displaying more activity of the sample used in the experiment when using 0.5 g catalyst.

Although there was a slight increase in long chain hydrocarbon production when using 0.5 g catalyst, as well as showing that there is more conversion at higher loadings, it is not significant enough to justify the extra material. This case for the highest catalytic loading further weakens as the results of using 0.35 g of catalyst are comparable to the 0.5 g loading. Furthermore, this study also finds that this reaction is more responsive toward temperature and reaction time.

### Influence of Reactants Ratio

The final series of experiments into conditions' optimization concerns the effect that varying the reactant ratio has on the system. This study was conducted with the reaction time (18 h), catalyst amount (0.5 g) and the temperature (300°C) remaining constant. The typical molar ratio of the reactants, which has been used in all of the studies presented this far, is designed to mimic the ratio produced from sugar fermentation: A:B:E 3:6:1 (Cabezas et al., 2019). Three experiments were conducted: K1 has increased ethanol, while keeping the other two constant compared to the typical ratio. This produced a reactant molar ratio of 3:6:2. In more detail, the moles of ethanol used in the original experiment was doubled (0.2054 moles). The next experiment, K2, used a ratio with decreased acetone, compared to the typical ratio while maintaining ethanol and butanol, producing a molar ratio of 3:12:2 i.e., the moles of acetone in the original experiment were halved (0.153 moles). Finally, the third experiment, K3, involved the reduction of butanol; keeping the other two components of the mixture constant, to achieve a molar ratio of 3:3:1 through halving the moles of butanol when compared to the original experiment (0.3 moles). As mentioned, the basis of this study is the typical molar ratio, so all changes are made in comparison to that ratio while keeping the others as they were in the typical molar ratio of 3:6:1.

The impact of the reactant ratio variation is found in the conversion values presented in Table 7 as well as yield (Table 8 and Figure 12).

**Table 7:** Conversion of the reactants to products using the Fe catalyst at varied reactant molar ratios with constant reaction time, temperature, and catalyst amount.

**Reactant ratio**	**Conversion (%)**		
	**Acetone**	**Butanol**	**Ethanol**
3:6:2 (K1)	99.6	–	97
3:12:2 (K2)	98.6	–	94.8
3:3:1 (K3)	99	88.3	94.4
3:6:1 (Original)	99.6	95.9	95.2

**Table 8:** Yield of products having highest concentration with various reactant ratios using the Fe catalyst with constant amount, reaction time, and temperature.

**Reactant ratio**		**3:6:2 (K1)**	**3:12:2 (K2)**	**3:3:1 (K3)**	**3:6:1 (Original)**
Yield (%)	2-Propanol (C_3_)	9.9	6	17.6	6
	Butanal (C_4_)	0.8	0.94	1.3	2
	2-Heptanone (C_7_)	4.3	5	7.4	3.8
	2-Heptanol (C_7_)	0.6	0.6	8.2	0.64
	3-Hepten-2-one (C_7_)	0.4	0.5	0.8	0.5
	Butylbutyrate (C_8_)	0.35	0.5	0.54	0.4
	Isophorone (C_9_)	0.3	0.2	0.65	0.34
	6-undecanone (C_11_)	0.41	0.5	0.64	0.4

**Figure 11:**
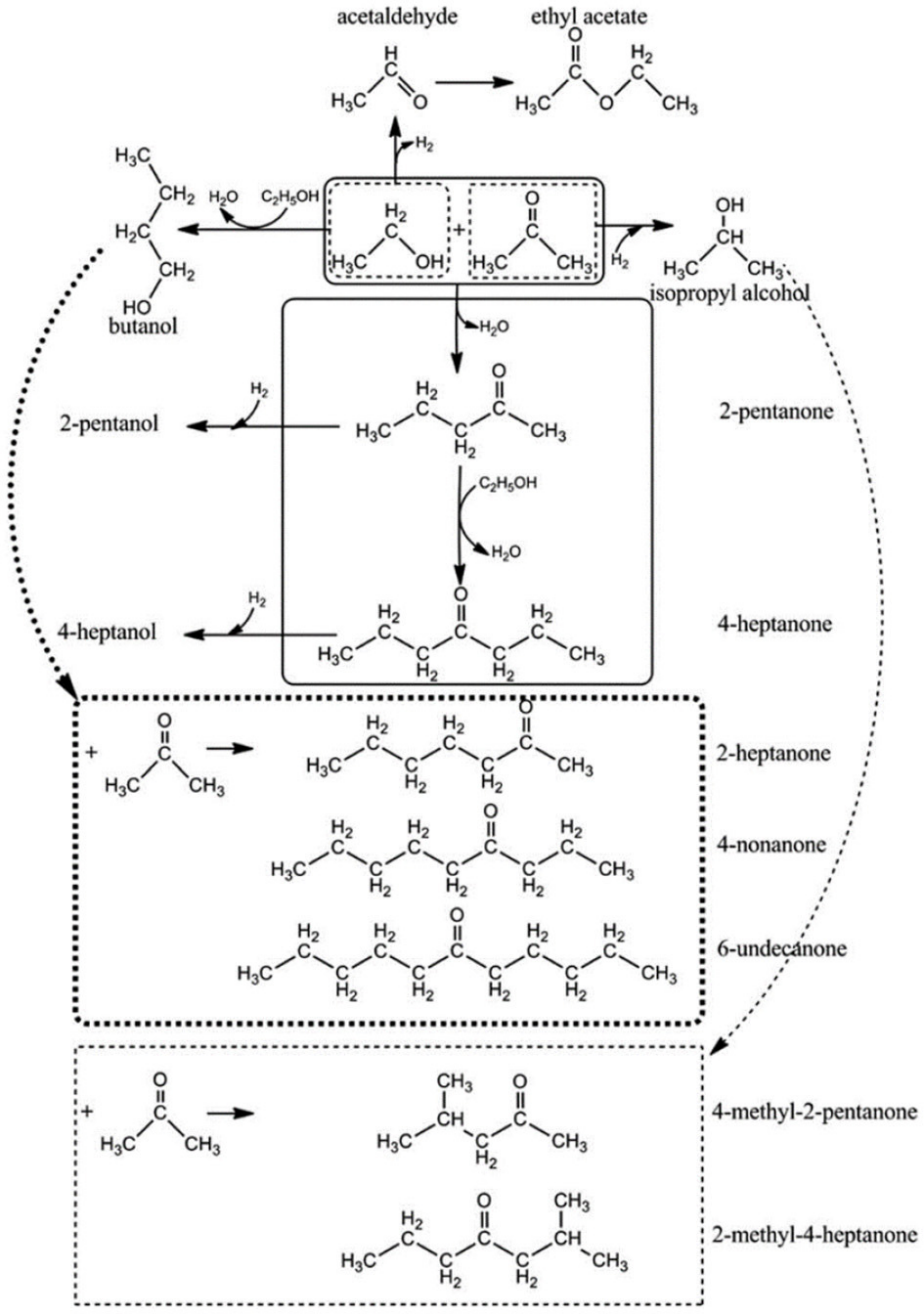
Possible reaction route between acetone and ethanol (Onyestyák et al., 2015a). Published by the Royal Society of Chemistry.

**Figure 12:**
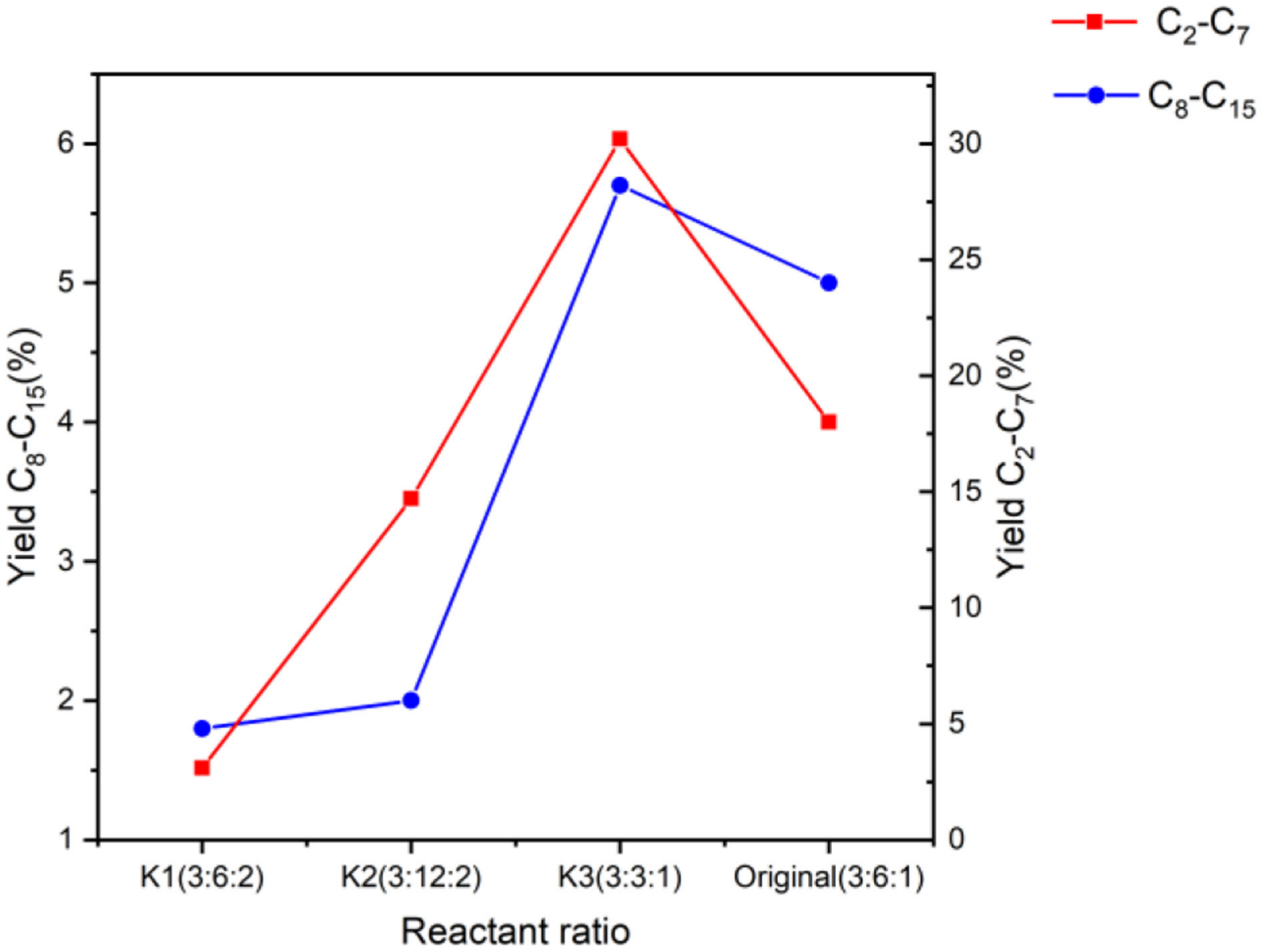
Yield of products in the range of C_2_-C_15_ using the Fe catalyst at various reactant amounts keeping the reaction time, temperature, and catalyst amount constant.

Starting with experiment K1, high conversions of acetone and ethanol can be seen and similar to the previous tests, butanol conversion cannot be calculated which could be also explained via the reaction mechanism, due to the increase of ethanol that results in an increase in butanol intermediates (Figure 3). In terms of yields, an increase in ethanol has led to lower yields for both classifications of hydrocarbons. This could be explained through the role of ethanol within the reaction network as the precursor for many of the initial reactions, i.e., ethanol dehydrogenation through the basic site of the catalyst to produce acetaldehyde presented in Figure 11 (Onyestyák et al., 2015b). This aldehyde has been shown to have a limited role as an intermediate in the reaction as well as its production being the limiting step for the reaction network. Previous studies have also found that acetaldehyde does not show significant contribution to the carbon chain growth (Ndou et al., 2003). Therefore, it follows that the increase of ethanol in the reactant mixture increases the production of acetaldehyde, leading to the limitation of reaction progress and resulting in the lower yields seen in Figure 12.

It should be also noted that due to the volatility of acetaldehyde, this product would be in the form of gas at room temperature and as such, would not be identified in this study as only the liquid products were analyzed. This would explain the drop in the yield of the short chain range of hydrocarbons presented in this experiment.

The next experiment (K2) shows promising results in terms of acetone and ethanol conversion, though any data regarding butanol is impossible to determine, owing to the imposed excesses used in the reactants and obtained in the products. Comparing these results to the original molar ratio, the conversion has increased but the yield for both ranges of products is less.

Experiment K2 (3:12:2, A:B:E) was undertaken with an acetone deficient from the original ratio. This leads to butanol excess in the whole system as can be seen in the ratio in comparison to acetone and ethanol. This reduction of acetone resulted in an increased yield of both hydrocarbon classifications when compared to K1 but still less than the original molar ratio. Decreasing acetone in the system reduces the reactions involving acetone, as expected. For example, there will be less reactions of butanol and acetone (Figure 11) that results in 2-heptanone and heptanol or less reactions of all three reactants together that ultimately results in long chain hydrocarbons such as 4-nonanone and 6-undecanone (Breitkreuz et al., 2014), which clearly explains the reduction in long chain production.

Finally, experiment K3 demonstrates considerable levels of conversion for ethanol and acetone, in addition to high conversion of butanol, which can be calculated as there is less butanol in the system as this ratio utilizes less butanol in the system, having the molar ratio of 3:3:1. This makes the ethanol and butanol content an equal ratio in comparison to acetone. This has led to significantly higher yields for the C_2_-C_7_ range, which supports the role of butanol in the production of longer chain hydrocarbons.

When compared to a significant relative increase in butanol content, such as the original ratio, we can speculate that this surplus would favor butanol dehydration that would possibly occur in a complex mechanism according to Nahreen and Gupta (2013). This reaction would result in the production of either alkenes or ethers depending on the reaction temperature. Nahreen and Gupta have stated that at temperatures higher than 300°C, olefin production is favored, while at lower temperatures ethers are the main product (Nahreen and Gupta, 2013). Considering the reaction temperature in this study, the main product should be ethers, which are significantly less reactive than alkenes. This leads to less progression of the reaction and prohibiting the elongation of butanol to longer chain hydrocarbons such as 2-heptanone, 2-heptanol, 6-undecanone, etc.

Therefore, the increase of the aforementioned products when conducting experiment K3 can be seen in Table 8, a testament to the effect of having less butanol in the system.

The XRD patterns not shown for sake of briefness reveal the presence of MgO, Al_2_O_3_, and Fe can be seen in the spent samples alongside emerging Fe_2_O_3_ peaks (Malinowski et al., 2009; Strassberger et al., 2013; Wang et al., 2014; Dasireddy et al., 2018) for the same reasons explained above—partial oxidation of iron under hydrothermal conditions.

The peak intensity of Fe at 44.7° in the spent samples (compared to the reduced sample), details reduction. However, comparing the main peaks for K1-3 and the original sample, the Fe_2_O_3_ peak at 35.4° for K3 has the highest intensity suggesting the highest oxidation compared to the others which is in line with fact that K3 has shown the highest selectivity and activity.

**Figure 13:**
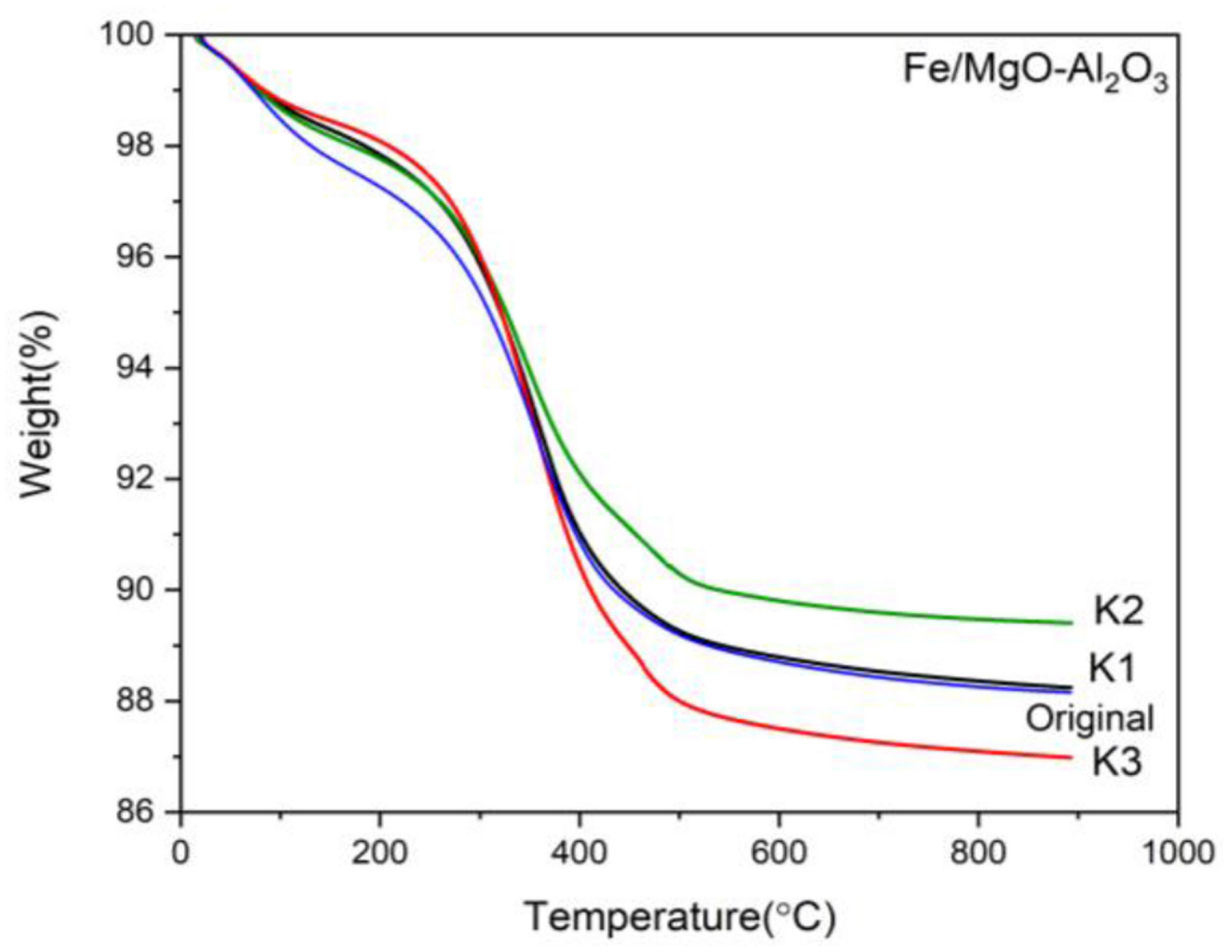
TGA curve of spent Fe catalyst at various reactant molar ratios.

The yield and conversion results are also in good agreement with the TGA presented in Figure 13, showing that the most loss associated with organic compounds is for experiment K3 for both light and heavy organic compound zones (150–350 and 350–500°C, respectively), which also presents the highest yield and conversion. The least amount of carbon is deposited on K2 even though the yield is higher than K1. That could be due to the fact that we have only analyzed the liquid and the main product of K1 is considered to be acetaldehyde, which was in the form of gas hence not detected. We can therefore conclude that the most activity was achieved with the K3 ratio, having the highest weight loss (11.5%), due to the most organic compounds attached.

Comparing K1–3 to the original, it is clear that K3 has higher yields but less conversion of butanol when compared to the original experiment. Nevertheless, the improvement (especially for the long chain hydrocarbons) is not significant enough to become the new “standard” conditions for this process; it should be taken into account that the original molar ratio is naturally produced via sugar fermentation and to change that ratio would require time and expense to enrich the feed, rather than instantly utilizing the natural product ratio from sugar fermentation.

### Catalyst Recyclability

**Figure 14:**
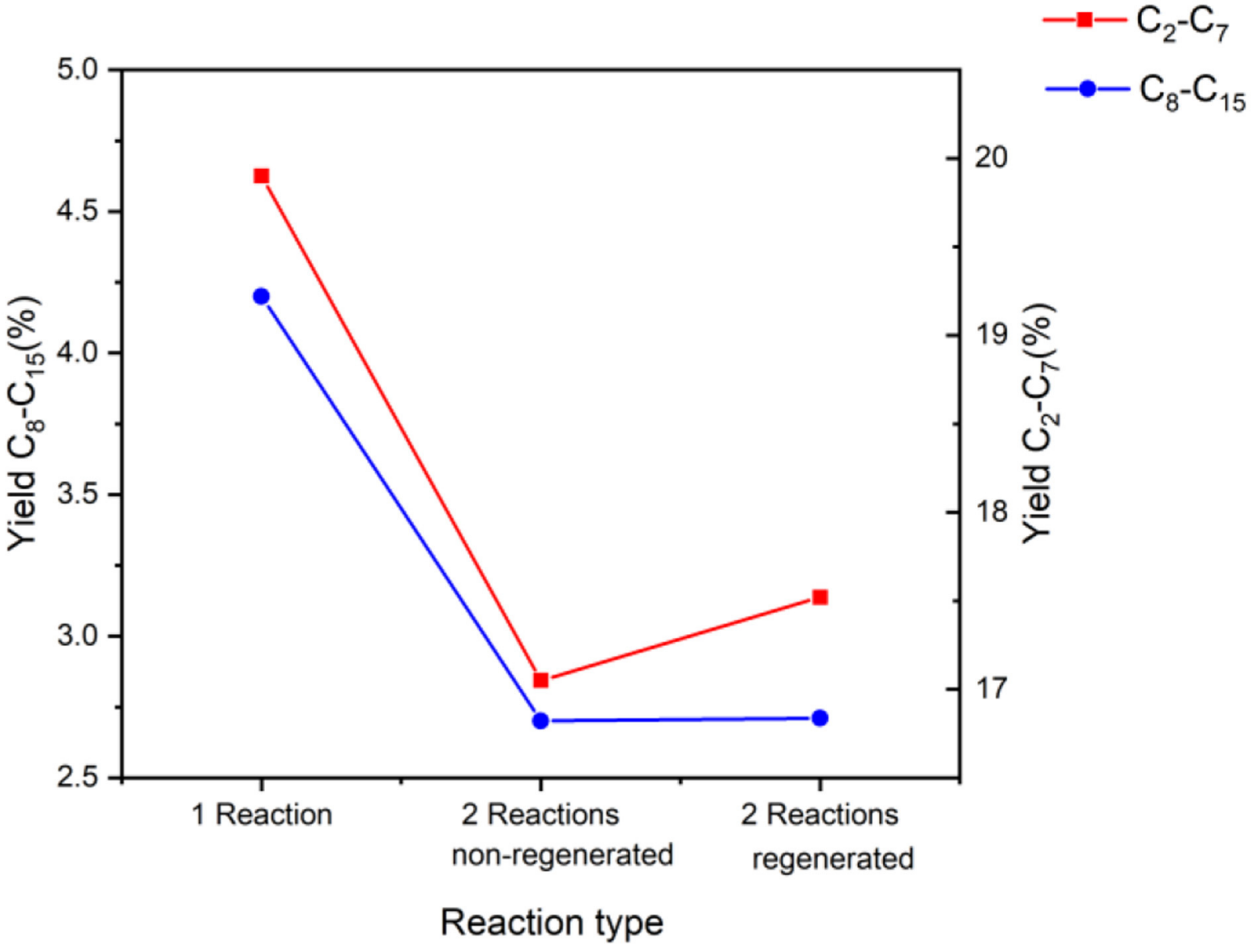
Yield of products in the range of C_2_-C_15_ using the Fe catalyst, comparing standard reaction with re-used catalyst with and without activation.

Catalyst's lifespan and recyclability are vital for practical applications. Toward this end, two experiments were devised that each involved 2 reactions. The first experiment (non-regenerated study) introduced the catalyst sample as recovered from the first reaction, in a second reaction. The second experiment (regenerated study) proceeded in the same way but used a reduction step for the catalyst prior to the second reaction. The conditions for both reactions were the same as used previously: 0.35 g catalyst, 3:6:1 ABE molar at 300°C, aiming to use the optimized conditions. Both the catalyst samples are reused only once, directly after drying in a reaction with the same conditions as the initial experiment. The catalytic activity results of both studies can be found in Tables 9, 10 as well as Figure 14.

**Table 9:** Conversion of the reactants to products using the Fe catalyst comparing standard reaction with re-used catalyst with and without activation.

**Reaction type**	**Conversion (%)**		
	**Acetone**	**Butanol**	**Ethanol**
1 Reaction	99.6	–	95.1
2 Reactions (non-regenerated)	99.6	–	94.4
2 Reactions (regenerated)	100	–	95.1

**Table 10:** Yield of significant products when using the Fe catalyst recycled without regeneration, recycled with regeneration as well as the standard reaction chosen for comparison.

**Reaction type**		**2 Reactions (Non-regenerated)**	**2 Reactions (Regenerated)**	**1 Reaction** **(0.35 g_**cat**_ catalyst, 300^**°**^C, 18 h)**
Yield (%)	2-Propanol (C_3_)	9.3	6	7
	Butanal (C_4_)	0.95	0.9	0.9
	2-Heptanone (C_7_)	4.8	4.7	5
	2-Heptanol (C_7_)	0.6	0.6	0.6
	3-Hepten-2-one (C_7_)	0.5	0.5	0.5
	Butylbutyrate (C_8_)	0.34	0.3	0.4
	Isophorone (C_9_)	0.3	0.2	0.33
	6-undecanone (C_11_)	0.4	0.3	0.4

Starting with conversion, Table 9 displays these values which prove the success of the catalyst even when re-used in both cases.

When analyzing the yields obtained in this set of experiments, it is clear that there is a minor decrease after re-using the catalyst (Figure 14). However, the values are comparable with the one reaction experiment (original reaction), justifying the reliability of the catalyst even after recycling. Comparing the regenerated and non-regenerated experiments, the addition of the reduction step shows a minor improvement in product yields, though not enough to demand the adoption of a reduction step prior to recycling (only 2.7% improvement). This trend is also seen in the significant product yields (Table 10). Here it can be seen that the significant product yields are slightly higher in the one reaction experiment, as can be expected, but nevertheless the difference is very minor. We do however see that in the case of 2-propanol, its yield is at its highest when recycled without reduction. This could mean that the catalyst was able to produce this product but as it was not able to further elongate this to long chain hydrocarbons, it has higher yield compared to the others.

**Figure 15:**
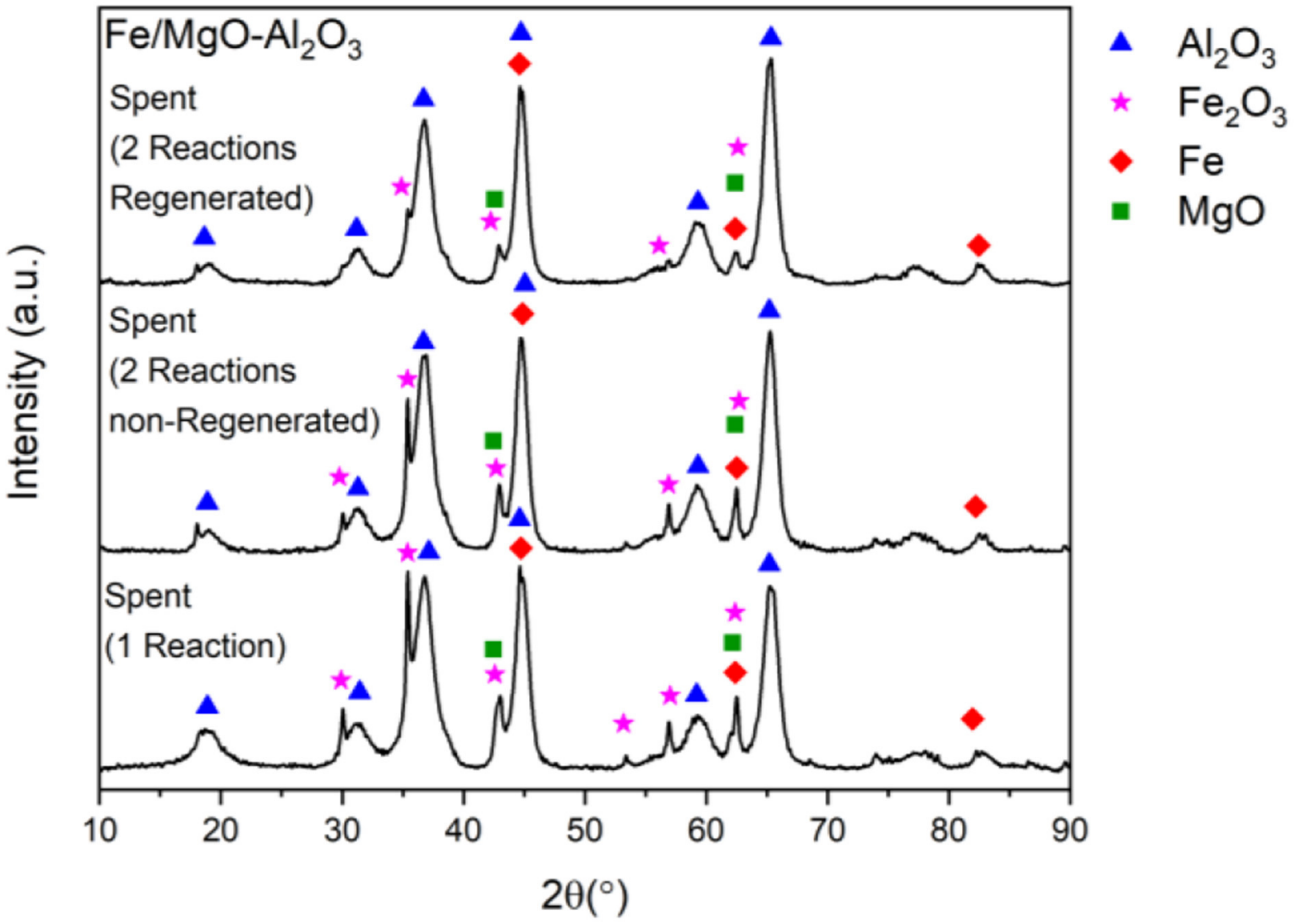
XRD of spent Fe catalyst non-recycled, recycled without activation and recycled with activation.

As can be seen in Figure 15, the patterns for the spent samples of the non-regenerated and regenerated studies are compared to an experiment where no recycling has taken place. Consistent peaks of the support and Fe can be seen in all diffractograms with the inclusion of small Fe_2_O_3_ peaks, which decrease, in the case where the sample was re-reduced. The main Fe peak remains the same when comparing the spent sample with all recyclability studies. The oxidized Fe peak is also maintained when comparing first experiment and second (which includes both reduced and non-reduced), which is a testament to the stability of the catalyst even after re-use.

### Simplified Preliminary Economic Assessment

Continuing toward the aim of improving energy consumption and cost, a simplified preliminary economic analysis of the reactants and significant products has been conducted to display the added market value of the products, compared to the starting components. The costings have been conducted with respect to the ratio of ABE used in the reaction (3:6:1) alongside the amount of significant products produced at the reference conditions we have used in this study: 300°C, 18 h and 0.5 g catalyst.

Tables 11, 12 showcase the amounts of the components, as well as their price, followed by the price per kg of material for both the reactants and products.

**Table 11:** Pricing of reactants used in ABE upgrading process (Merck, 2019a,b,c).

**Reactants**	**Input (Kmoles) X 10^**−3**^**	**Input (Kg) X 10^**−3**^**	**Price (US$/Kg)**	**Price for reactant for this process** **(US$/Kg input)**
Acetone	0.306	18	2.55	45.32
Butanol	0.601	44	1.22	54.35
Ethanol	0.103	5	0.89	4.21
Total	1.009	67	–	103.87

**Table 12:** Pricing of significant products obtained from ABE upgrading process (Alibaba.com, 2019; Merck, 2019d).

**Significant products**	**Output** **(Kmoles)** **X 10^**−3**^**	**Output** **(Kg) X 10^**−3**^**	**US$/Kg**	**Price for product** **for this process** **(US$/Kg input)**
2-Propanol	0.07	4	1.60	6.73
Butanal	0.017	1	2.50	3.06
Butyl acetate	0.0016	0.19	1.00	0.19
2-Heptanone	0.0772	9	5.00	44.07
2-Heptanol	0.0074	0.86	25.00	21.49
Butyl butyrate	0.0022	0.32	56.75	18.00
3-Hepten-2-one	0.0024	0.27	2158.50	581.08
Isophorone	0.00013	0.2	1.30	0.023
6-Undecanone	0.0076	1.3	30.00	38.827
Total	0.1855	17.14	–	713.49

Comparing the two tables, it can be seen that there is approximately an 85% price increase from reactants to products, demonstrating the profit obtained when analyzing the prices of the starting material and end material, considering the amount of reactants used and yields obtained. This is another proof of the favorability of this process, not only providing a large number of different organic compounds that are suitable for chemicals and/or transportation industry, but also having market value much higher than the starting chemicals. It is evident that for attaining practical economic profit and benefit presented above, scaling up is key in order to obtain higher concentrations of the products. Nevertheless, lab scale results demonstrate an 85% price increase, an increase that would still be present in larger scale instances, making this a very attractive route of chemicals and/ or bio-fuel production.

It must be noted that the separation cost of the end products as well as the utilization of real feedstock including for example water is not considered in this study since this is beyond the scope of this proof-of-concept paper, though the profit margin is so high that it remains economically favorable.

## Conclusions

The catalytic upgrading of Acetone/Ethanol/Butanol mixtures from sugar fermentation has been successfully demonstrated in this study using a highly effective Fe/MgO-Al_2_O_3_ catalyst which has evidenced not only high levels of conversion and yield toward upgraded products but also remarkable levels of stability via recycling tests. The effect of reaction parameters on a complex reaction scheme, which involves primarily aldol condensation reactions, have been investigated in this study aiming to discern the most favorable conditions. The effects of temperature, reaction time, catalyst loading, and reactant molar ratio have been investigated through systematic testing to observe the changes incurred by each variable while also targeting the reduction of energy, time, material and costs. Varying the temperature for this reaction found the reaction scheme to perform as expected for an endothermic system, performing best at the highest temperature (300°C) in terms of yield and conversion. However, catalyst oxidation can be seen on the XRD results at temperatures >250°C, though this did not appear to impede the catalyst activity. After testing the effect of reaction time, it is clear that the 18 h test was able to convert the reactants to the desired longer chain products.

Comparing results from the temperature and the reaction time studies, it appears that higher temperatures play a more prominent role in the yielding of longer chained hydrocarbons than the reaction time. This was exemplified when comparing the lowest temperature experiment with the highest reaction time. Even though the 200°C experiment had an 18 h reaction time, the reaction produced lower amounts of C_7_ hydrocarbons than at 300^o^C. This higher temperature reaction at only 3 h of reaction time managed to produce hydrocarbon chains up to C_11_. This clearly indicates that the reaction performs best at higher temperatures rather than longer reaction times.

The influence of the reactant ratio highlighted the importance of butanol in the mixture, showing that the ratio of butanol in comparison to the other two components has led to the biggest impact on product yield. This is seen in the K3 experiment, which used the reduced butanol component (3:3:1, ABE) compared to the typical ratio, the yield is higher than the original experiment (3:6:1, ABE). This resultant enhancement to yields is not, however, enough to justify the further processing that the ABE molar ratio obtained from sugar fermentation would require.

Finally, we have also proven the viability of this process through a simplified preliminary economic analysis, showcasing the increased market value of the significant products of the ABE upgrading. Also, the authors sympathize with the limitations of this seminal study including, for example, the need to use a real mixture for the process which includes water and also the further downstream separation processes but, in any case, the obtained results are encouraging. Hence, we hope this work sparks further research within the catalysis community to develop the next generation low-carbon technologies via economically appealing processes using biorefinery inlet feedstock to produce added value chemicals.

## Data Availability Statement

The XRD and TGA datasets generated for this study can be found in Dryad at https://doi.org/10.5061/dryad.5dv41ns2z.

## Author Contributions

TR, LP-P, and HA-G contributed conception and design of the study. EK conducted the reactions, characterization, and analyses of the data. EK, LP-P, and TR wrote sections of the manuscript. All authors contributed to manuscript revision, read, and approved the submission version.

### Conflict of Interest

The authors declare that the research was conducted in the absence of any commercial or financial relationships that could be construed as a potential conflict of interest.
